# Priming in word stem completion tasks: comparison with previous results in word fragment completion tasks

**DOI:** 10.3389/fpsyg.2015.01172

**Published:** 2015-08-13

**Authors:** María J. Soler, Carmen Dasí, Juan C. Ruiz

**Affiliations:** Faculty of Psychology, University of Valencia, Valencia, Spain

**Keywords:** implicit memory, priming, word fragment completion, word stem completion

## Abstract

This study investigates priming in an implicit word stem completion (WSC) task by analyzing the effect of linguistic stimuli characteristics on said task. A total of 305 participants performed a WSC task in two phases (study and test). The test phase included 63 unique-solution stems and 63 multiple-solution stems. Analysis revealed that priming (mean = 0.22) was stronger in the case of multiple-solution stems, indicating that they were not a homogeneous group of stimuli. Thus, further analyses were performed only for the data of the unique-solution stems. Correlations between priming and familiarity, frequency of use, and baseline completion were significant. The less familiar words, which were less frequent, had higher priming values. At the same time, the stems with lower baseline completion generated more priming. A regression analysis showed that baseline completion was the only significant predictor of priming, suggesting that the previous processing of the stimuli had a greater impact on the stimuli with low baseline performance. At the same time, baseline completion showed significant positive correlations with familiarity and frequency of use, and a negative correlation with length. When baseline completion was the dependent variable in the regression analysis, the significant variables in the regression were familiarity and length. These results were compared with those obtained in a study using word fragment completion (WFC) by Soler et al. (2009), in which the same words and procedure were employed. Analysis showed that the variables that correlated with priming were the same as in the WSC task, and that completion baseline was the variable that showed the greatest predictive power of priming. This coincidence of results obtained with WFC and WSC tasks highlights the importance of controlling the characteristics of the stimuli used when exploring the nature of priming.

## Introduction

During the last few decades, implicit memory research has employed a variety of tests to elicit unconscious memory (Capner et al., 2007). Some of these tests analyze priming, defined as an effect in which exposure to a stimulus influences response to a later stimulus (Hsu and Schütt, 2012). Due to the diversity of the implicit tasks used in the study of priming, different classifications have been proposed in an attempt to clarify exactly which processes are involved in each one and, consequently, the nature of the priming effect in the different tasks. The classification proposed by Toth (2000), which has been supported by empirical research analyzing behavioral responses or performance, and by data obtained using neuroimaging techniques (Schacter et al., 2007), identifies perceptual and conceptual measures, among other tasks. Perceptual tasks, such as the word fragment completion (WFC) task, require stimulus processing based on their physical characteristics, and priming is widely accepted to reflect facilitation of perceptual processes in these tasks (Roediger and McDermott, 1993; Rueckl and Mathew, 1999; Kinjo and Snodgrass, 2000; Blum and Yonelinas, 2001). Conceptual tasks, such as the word stem completion (WSC) task, elicit responses based on the retrieval of aspects of stimuli meaning (Blaxton, 1989; Rueckl and Mathew, 1999; Ryan et al., 2001; Mitchell and Bruss, 2003; Fleischman et al., 2005). However, the nature of priming in WSC tasks is unclear and is the subject of controversy (Roediger et al., 1992; Bruss and Mitchell, 2009; Brooks and Gibson, 2012). For example, Roediger et al. (1992) analyzed the differential effects of some variables in WFC and WSC tasks and did not find dissociative effects on priming. This led them to conclude that the priming generated by the WSC task is the same as that of the WFC task and that priming has a perceptual nature in both cases. Bruss and Mitchell (2009) performed a factor analysis and categorized the WFC task as perceptual, while the WSC task (with pictures) was included in the conceptual implicit memory tasks group, but they concluded that the latter task (with words) is a more complex measure that involves the interaction of different processes or systems. Using a similar approach to explain priming, Brooks and Gibson (2012) found that both tasks loaded on the same factor, but that WFC had a negative loading. The authors suggested that the explanation for these findings should go beyond whether they are perceptual or conceptual tasks, and should focus on analyzing the cognitive resources that cause an overlap of prime and task demands. In line with this proposal, the distinction between implicit tasks based on identification processes (e.g., WFC) and those based on production processes (e.g., WSC) (Gabrieli et al., 1999; Spataro et al., 2011) is also a factor that requires consideration.

In summary, the debate is still open. In the light of the data we have obtained it is reasonable to think that some characteristics of the tasks are methodologically problematic and may introduce some noise in the controversy. For example, according to Roediger et al. (1992), one possible explanation for the contradictory results observed when WFC and WSC tasks are compared is that most of the characteristics of the stimuli used in each one are usually different. They highlighted that different studies have shown that the WSC task is solved more easily and faster than the WFC task because researchers usually select stems corresponding with short, frequently used words and with multiple solutions (often more than 10); in such conditions, subjects run a high probability of identifying one of the possible answers. However, in WFC tasks, the fragments are usually based on long, less frequently used words and have only one or two possible solutions, which means that the task is more difficult and requires more effort. As a consequence, participants are not always able to find an appropriate response to the fragment. In line with the observations of Roediger et al. (1992), it could be relevant to review how variables such as number of possible solutions, or frequency and length of the words used as stimuli in the tasks, influence the probability of correct completion of a stimulus or baseline completion and priming scores.

Some studies have shown that, in implicit tasks, when there is only one possible solution, such as in WFC tasks, the effect of the study phase on low baseline completion stimuli is higher (Scarborough et al., 1977; Ostergaard, 1998, 1999). If the fragments have high baseline, the probability of correct completion is high, even if they have not been processed during the study phase. Thus, the effects on priming of the experimental conditions set up in the WFC tasks could be canceled because the task is easy. In fact it has been observed that the effect of variables such as word frequency on priming only appear when the baseline is low (Ostergaard, 1998). Similarly, in WSC tasks that use multiple solution stems, a low target baseline implies that other correct responses will have higher probabilities of being selected. In this case, a single presentation of the stimulus during the study phase may not be enough for the subject to be able to choose it as a response during the test phase. However, if the target has a high baseline, one presentation during the study phase may be sufficient to make it more accessible in comparison with other competitor responses (Ryan et al., 2001). Considered together, these results suggest that it is necessary to control the baseline of the stimuli when exploring experimental effects on priming.

There is a second factor that should be considered when comparing the results of WSC and WFC tasks; namely, the number of solutions for stems and fragments. Studies with WSC tasks frequently use multiple solution stems, and studies with WFC tasks usually use fragments with one solution. For example, Roediger et al. (1992) used unique solution fragments, but all their stems had multiple solutions, while Craik et al. (1994) used fragments with unique solutions and stems with at least four solutions. Some authors have suggested that having one or multiple response alternatives means that the cognitive resources that overlap the presentation of prime and task demands are different in both tasks (e.g., Gabrieli et al., 1999; Spataro et al., 2011). This is the reason why the WFC task is assumed to be an identification task. Participants are required to identify stimuli characteristics: first, they must analyze the stimuli form; second, they must identify the only correct response among all the possible solutions in the recovery process. The WSC task is classified as a production task because the stem presented to the participant serves as a cue to elaborate different candidate responses, and to initiate, later on, a recovery process in which there is competition between the candidates. Research exploring the effect of number of solutions on priming and baseline in WSC tasks is limited. In one of the few studies carried out (Gibson, 2012, Experiment 3), priming obtained with unique solution and with more than 10 solution stems was compared and results indicated that there were no differences. However, an interesting finding of that experiment was that baselines were lower in the case of unique solution stems, indicating that the probability of correct completion of a stem is greater when there is only one correct possible solution. In the study in question the frequencies of the different correct alternatives were found to be the same. This is relevant, as in unique solution stems, it could be hypothesized that the baseline is a measure of the probability of choosing the alternative selected by the researcher, and not a measure of the difficulty of the task (Ostergaard, 1998). In summary, the number of possible solutions should be considered a relevant variable in studies comparing WSC and WFC tasks, as it has an effect on the baselines of the targets and could have also influence the measurement of priming magnitude.

Finally, a set of variables related with the linguistic characteristics of the words from which stems and fragments are built should also be considered. According to the information variability model of Ostergaard (1998), the information accumulated in the memory from previous encounters with a word (e.g., frequency or familiarity) is used to solve tasks in which that word has to be identified. There is evidence indicating that priming and probability of stem or fragment completion correlate with a set of word linguistic characteristics (Balota and Chumbley, 1984; Erickson et al., 1987; Graf and Williams, 1987; Olofsson and Nyberg, 1995). Erickson et al. (1987) and Olofsson and Nyberg (1995) found that word familiarity is one of the factors that explains baseline performance in WSC and WFC tasks. Their data showed a positive correlation between familiarity (measured with a subjective scale) and target completion probability. Targets corresponding to more familiar words are more easily completed than targets corresponding to less familiar words. Graf and Williams (1987) found positive correlations between word frequency, number of meanings of the word, and stem completion probability, and negative correlation between word length and baseline, and between word length and frequency. This last result suggests that, in the case of short words, there is a stronger codification among the letters that form the word than between the letters that form longer words. Recently, Mueller and Thanasuan (2014) have confirmed that the stems of frequently used words are easier to complete, suggesting that this is due to a stronger association between the letters within the word than that in less frequent words. When priming has been assessed, results show that frequency is one of the variables that explain priming scores. Studies with WFC and WSC tasks have demonstrated that priming scores are lower in high frequency words than in low frequency words (MacLeod, 1989; Roediger et al., 1992; MacLeod and Kampe, 1996; Soler et al., 2002), and that less familiar words produce higher priming values (Soler et al., 2009). These results could be explained by the fact that, in the case of the targets of low frequency and low familiarity words, the information available in the memory is scarce and, as a consequence, the impact of the processing of the word during the first phase of the task is strong. However, in high frequent and high familiar words, the relative increment in the information available in the memory due to a new encounter with the word would be small.

In summary, the selection of the stimuli and the control of potentially confounding variables should be considered when attempting to understand the differences between the results of studies comparing WFC and WSC tasks to infer valid conclusions about the nature of these tasks. In our research, these criteria have been considered in order to achieve two main objectives. First, to examine the effect of the following factors on priming in WSC: (1) attributes of stems such as baseline completion and number of solutions; and (2) linguistic attributes of words such as frequency, familiarity, number of meanings and length. Secondly, we aimed to compare these results with part of the data obtained in a previous study with WFC tasks published by Soler et al. (2009). In order to equate the two tasks as much as possible we designed an experiment using: (a) a WSC task with constrained-length stems; (b) the same codification instructions for the stimuli for all the participants; (c) stems generated from the same words used to build the fragments; and (d) two types of stems (unique-solution and multiple-solution).

## Materials and Methods

### Participants

A total of 305 undergraduate students enrolled in the University of Valencia’s introductory psychology courses participated in the study. All were native Spanish speakers in the age range of 19 to and 42 (mean = 21.71), and had normal vision or vision corrected to normal. All participants gave their written informed consent prior to participation, after having had the procedures explained to them. The study was in line with the Helsinki Declaration.

### Materials and Procedure

The stimuli used in this experiment were obtained from the Soler et al. (2009) database. This database contains a total of 269 words related with the following indices, among others: number of meanings, frequency (number of occurrences per million), and familiarity (estimation of the frequency of the use of a word, measured on a 7-point scale). This database also contains a unique-solution word fragment for each word, and the following indices related with the fragments: baseline of completion (indicates the probability that a fragment will be correctly completed); priming (increase in the proportion of correct completions of a fragment when its corresponding word has recently been processed); and ratio of letters to blanks (number of given letters divided by the number of deleted letters). The fragments were prepared by randomly deleting some letters from each original word. Two letters were deleted from five-letter words, two or three letters were deleted from six-letter words, and three or four letters were deleted from seven-letter words, following the Rajaram and Roediger (1993) method. Each fragment was then checked until a single correct solution fragment was confirmed. Baseline and priming indices of 269 database fragments were obtained in a two-phase fragment completion task. The first was the study phase, in which participants had to assess the familiarity of a pool of words. In the second phase, participants had to complete a list of fragments, half of which came from words presented in the first phase. Between the study and the test phase, participants performed a distracting task for a 5 min period. The task was implicit because no reference to the study phase was made in the test phase.

To fulfill the main objectives of this work a constrained-stem was built for each one of the 269 words from the Soler et al. (2009) database. The three first letters of the word composed each stem and the remaining letters were replaced by underscores or blanks (Gibson, 2012). A correct solution was any singular noun with the same number of letters as the corresponding word and a frequency of use higher than 0. For each stem, all the possible words fitting the stem were located in the Dictionary of the Spanish Language (Royal Spanish Academy of Language, 2001). Given that we wished to compare priming in WFC vs. WSC, and taking into account that all the fragments had a single correct solution, we first selected stems with only one correct solution (*n* = 63). We then added a new group of 63 multiple solution stems (with 3 to 6 solutions, mean = 4.14, SE = 1.13) in order to test the effect of number of solutions on priming. Therefore, the total amount of stimuli was 126.

The WSC task in our study was conducted in two phases following the procedure described above and used in a previous study on WFC (Soler et al., 2009). In this way, we aimed to avoid the effects that changes in the procedure could have on the magnitude of priming. In the study phase, a set of 45 words (all in lower case) was presented in the center of the computer screen for 8 s each. Participants had to judge and score on an answer sheet the familiarity of each word on a scale ranging from 1 (unfamiliar word) to 7 (very familiar word), according to the procedure of Erickson et al. (1987). In the test phase, participants were presented with a list of 90 stems in lowercase letters in the center of the screen for 12 s each. Half of the stems came from words viewed in the first phase of the experiment, and the other half came from non-viewed words. Participants were asked to write on the answer sheet the first word that came into their mind that corresponded with the set of letters and blanks in the stem. No explicit reference was made to the first phase of the experiment. Between the study and the test phase, participants performed a distracting task; namely, to write down the name of European cities during a 5 min period. Data were collected from small groups of 15 to 30 participants.

To avoid order effects, 6 lists of 90 stems were randomly selected from the 126 stimuli master list to be presented to participants in the test phase. The presence of all the stimuli in the lists was confirmed. Half of the stems corresponded with the 45 words presented in the study phase. The number of respondents for each list varied between 39 and 88, and so each word was not presented to an equal number of participants.

Following this procedure the average number of times each stem was presented for its completion was 51.81 (SD = 10.08). For each one of the lists a mirror list was built to interchange prime and target stimuli. The set of stems from words not seen in the study phase was used to obtain their baseline completion index. This index can be calculated in two ways (Table 1): (a) as a proportion of completions with the word used to build the stem; and (b), as a total proportion of correct completions. In a similar way to that used to calculate the baseline of each fragment in our database (Dasí et al., 2004, 2007; Soler et al., 2009), we employed the first option. This also served as the baseline to calculate priming (difference between baseline and proportion of correct completions when words have previously been presented).

**TABLE 1:** **Mean, standard deviation and skewness of the stimuli characteristics of the 126 words**.

**Variables**	***M***	**SD**	**Skewness**
Familiarity	4.85	1.41	–0.94
Frequency	42.86	81.25	4.98
Number of meanings	4.61	3.53	1.64
Length	5.96	0.76	0.07
Number of correct solutions of stems	2.57	1.77	0.60
Baseline completion rate of stems			
Unique-solution	0.57	0.32	–0.44
Multiple-solution^1^	0.32	0.25	0.59
Multiple-solution^2^	0.57	0.24	0.07
Priming of stems	0.22	0.15	0.50
Unique-solution	0.19	0.16	1.03
Multiple-solution	0.24	0.14	–0.06
Baseline completion rate of fragments	0.51	0.32	0.04
Priming of fragments	0.19	0.15	0.75

^1^Proportion of completions with the word used to calculate priming.

^2^Total proportion of completions.

## Results

Statistical analyses were performed for the data obtained in the WSC experiment. Additional analyses of part of the data from Soler et al.’s (2009) WFC experiment were carried out in order to compare both tasks. The main descriptive statistics of all the variables of the 126 stimuli used in the WSC task are shown in Table 1. The table also contains the values corresponding to the characteristics of the word fragments (baseline completion rate and priming) used in our previous study with a WFC task. We calculated the baseline completion rate for the multiple-solution stems using two procedures: (a) proportion of completions with the word used to calculate priming; and, (b) total proportion of completions.

First, the significance of priming in WSC was studied. The analysis showed that priming was significant for unique-solution stems [*t* (62) = 9.57, *p* < 0.001] and for multiple-solution stems [*t* (62) = 14.07, *p* < 0.001]. In the WFC study, the magnitude of priming was also found to be significant [*t* (125) = 14.25, *p* < 0.001]. Priming values of stems with a unique solution were compared with priming values of stems with two or more solutions using the independent groups *t*-test to test the hypothesis of competitive responses (Ryan et al., 2001). The result revealed significant differences [*t* (124) = 1.98, *p* < 0.05] in favor of multiple-solution (mean = 0.24, SE = 0.02) versus unique-solution (mean = 0.19, SE = 0.02) stems. Cohen’s *d* effect size was 0.33.

Given the differences detected between unique- and multiple-solution stems, only the former were used to compare the priming of stems and fragments, as the fragments had only one solution. A paired-sample *t*-test did not reveal significant differences between the two means (0.19 for stems vs. 0.17 for the 63 fragments corresponding to the stems with a unique solution) [*t* (62) = 0.89, *p* > 0.37]. Cohen’s *d* effect size was lower than 0.11.

The matrix of Pearson correlations among these variables was performed to analyze the pattern of relations between them, in order to determine the most important factors underlying the priming (Table 2). The variables can be clustered as follows:

**TABLE 2:** **Matrix of Pearson correlations between the variables for the 63 stimuli corresponding to the unique-solution stimuli**.

	**Words**			**Stems**		**Fragments**	
**Variables**	**2**	**3**	**4**	**5**	**6**	**5**	**6**
1. Familiarity	0.75**	0.27*	–0.09	0.62**	–0.40**	0.39**	–0.36**
2. LogFrec	–	0.48**	–0.18	0.42**	–0.37**	0.28*	–0.43**
3. Meanings		–	–0.11	0.15	–0.23	0.08	–0.13
4. Length			–	–0.26*	0.07	–0.23	0.06
5. Baseline				–	–0.58**	–	–0.47**
6. Priming					–		–

Meanings, number of meanings; LogFrec, logarithm of frequency; *p < 0.05, **p < 0.01.

(a) Variables related to the original words of the stimuli: familiarity, frequency, number of meanings, and length. The frequency was logarithmically transformed *log (1* + *x)* following the method Cuetos and Alija (2003) to eliminate the strong positive skew of the distribution of the variable.
(b) Variables related to stems and fragments: baseline, and priming.

Table 2 shows significant correlations of stem priming with familiarity, logarithm of frequency, and baseline. In the case of fragments, the variables related with priming were the same. It is important to underline that the three linguistic variables (familiarity, logarithm of frequency and number of meanings) were significantly correlated, while none of them was significantly correlated with length. The differences in the size of correlations between the two tasks were measured, and nine comparisons were performed (columns 5 and 6 for stems and fragments in Table 2). None of them were statistically significant. Only the difference between baseline-familiarity correlations (0.62 vs. 0.39) was marginally significant (*p* < 0.10).

For stems, the variables familiarity, logarithm of frequency, number of meanings, length, and baseline were included in a stepwise multiple regression to explore their capacity to predict the priming. The result revealed that only the contribution of baseline was significant (β = –0.46, *p* < 0.001), accounting for 21% of the variance of priming. Using the data of our previous study with WFC, a second stepwise regression analysis was conducted for the 63 fragments of the unique-solution stems. Again, the same variables were used as possible predictors of priming (familiarity, logarithm of frequency, number of meanings, length and baseline). This time, unlike in the stems analysis, two variables proved to be significant: baseline (β = –0.42, *p* < 0.001) and logarithm of frequency (β = –0.32, *p* < 0.001). Baseline explained 24.1% of the variance, and logarithm of frequency 9.0%.

Due to the relevance of the variable baseline of correct completions with respect to priming, we decided to perform more specific analyses. First, to ensure that our results were not due to variations in the baseline of stems and fragments, a paired-sample *t*-test was carried out to compare the baseline of the 63 unique-solution stems and fragments. The baseline average was 0.57 (SE = 0.04) for the stems and 0.57 (SE = 0.04) for the fragments; the difference was not significant (*p* > 0.95; effect size = 0.0004). Second, two stepwise linear regressions were calculated in order to predict the baseline of stems and fragments using the variables familiarity, logarithm of frequency, number of meanings, and length. The results showed that the variable entered in the first step was familiarity (*r*^2^ = 0.30, β = 0.54, *p* < 0.001) in the case of stems. In the second step, the variable length reached statistical significance with an increase in the explained variance of 3.7% (β = –0.20, *p* < 0.01). In the case of fragments, the results were similar; the most significant variable was familiarity (*r*^2^ = 0.10, β = 0.31, *p* < 0.001), and the second most important was length, with a 2.3% (β = –0.17, *p* < 0.05) increment in the explained variance. In all the regression analyses, the diagnosis of collinearity gave Variance Inflation Factor values of around 1, which indicated no collinearity problems between predictor variables.

## Discussion

The present study aimed to examine the effects of some word and stem characteristics on priming in WSC tasks and to compare these results with previous results obtained by Soler et al. (2009) with WFC tasks. These two objectives not only have theoretical and taxonomic relevance, but also empirical implications, as these tasks are widely used in different research contexts.

To reach our objectives we first assessed the capacity of the WSC-constrained task to elicit priming. In WSC tasks, participants are usually instructed to complete the stem with the first word that comes into their mind without knowing the number of letters in the target word. In our study we used a version of the WSC task in which the participants are aware of the number of letters of the target using underscores. The results showed a significant priming effect reflected in an increment in the proportion of stems completed when their corresponding word had been seen in the first part of the task, in comparison with the proportion of stems completed if their corresponding word had not been presented in the first part of the task. The difference between the two proportions, or priming (mean = 0.22), was similar to that reported in WSC-unconstrained tasks (e.g., Roediger et al., 1992; Ryan et al., 2001). Secondly, we analyzed the effect of number of stem solutions on WSC priming, as this variable is not usually controlled in priming studies, and there are few data available regarding how it influences results. We compared the magnitude of priming obtained in unique- and multiple-solution stem conditions. Difference in priming was significant, with priming being stronger in the case of multiple-solution stems. This result shows that, for multiple-solution stems, previous presentation of one of the possible solutions makes this solution more accessible during the test part of the task, thus increasing the probability of producing the target as a response. Our results show that, although the baseline for multiple-solution stems (mean = 0.32) was below that of the unique-solution stems (mean = 0.57), the previous processing of the word produced more priming in the case of multiple-solution stems. These results are not in line with those of other studies. For example, Gibson (2012) did not report differences in priming between unique and multiple-solution stems, although the baselines in both conditions were different (unique-solutions mean = 0.43; multiple-solutions mean = 0.13). One possible reason for the discrepancies with Gibson’s results is that she used stems with 10 possible solutions, while we used stems with between 3 and 6 solutions. In any case, the conclusions we can draw regarding the effect of the number of solutions on priming are limited, as there are few studies that have explored this effect in depth. However, as we have found differences in priming between unique and multiple-solution stems, all the conclusions concerning our two main objectives have been drawn purely on the results of the analysis of our data for unique-solution stems. This is because we wanted to compare the data we obtained in the WSC tasks with that obtained in our previous research with WFC tasks, in which fragments had only one solution.

In relation with our first goal, we initially studied the pattern of relations between the stimuli linguistic characteristics, and results showed the expected significant positive correlations between the variables familiarity, frequency and number of meanings. All these variables are word attributes that measure similar constructs. Correlations between these variables indicate that words with a higher number of meanings are used in more varied contexts and more frequently than words with a lower number of meanings. This is the reason why their subjective familiarity and frequency are higher than that of words with a lower number of meanings. At the same time, the fourth linguistic variable—word length—measures a different construct that does not have a significant correlation with the other variables, but gives information about the stimuli that could be relevant in the WSC-constrained task.

The analysis of how linguistic variables explain *baseline stem completion* showed that baseline completion correlated significantly with the word familiarity (measured in a subjective scale), which is in line with previous results (Erickson et al., 1987; Olofsson and Nyberg, 1995). Stems built from highly familiar words have a higher completion probability than those built from less familiar words. Baseline completion also correlates significantly and positively with word frequency. Stems corresponding to high frequency words were easier to complete than stems corresponding to low frequency words, in accordance with previous studies (Graf and Williams, 1987). In line with Ostergaard (1998), these correlations suggest that, when a stem has to be completed, the subject accesses the information about the word that has been accumulated in the memory during past encounters with it. Our results also confirmed the negative correlation between word length and baseline completion (Graf and Williams, 1987), supporting the idea that, in short words, there is a strong codification within the letters that form the word. Therefore, when a stem has to be completed, it has a higher capacity to evoke the whole word. Finally, regression analysis results showed that the most relevant variables explaining stem completion probability were familiarity and word length.

In the case of *priming*, the correlation analysis of the linguistic variables showed that familiarity and frequency were significantly correlated with priming. The correlation between familiarity and frequency with priming was inverse, the less familiar words, which were less frequent, had higher priming values. Ostergaard (1998) has hypothesized that this inverse correlation, in the case of WSC tasks, can be explained by the type of word processed during the first phase of the task. In the case of highly familiar words there is a lot of information available related with previous experience of the word. In the case of words with low familiarity, information from previous experience of the word is more limited; as a consequence, the processing of the word during the first phase of the task becomes more relevant in the later processing of the stem. However, the first order correlations that appeared in our analysis do not allow establishing a direct causal relation between familiarity and frequency with priming.

The correlation we observed between baseline completion and priming deserves special attention. This correlation is negative; that is, stems with small completion probabilities produce the highest priming values. This result is in accordance with those obtained in other implicit tasks in which there is only one possible correct response, such as in lexical decision or unique-solution WFC. In these tasks, the relevance of the previous processing of the stimuli has a greater impact on stimuli with low baseline completion (Ryan et al., 2001). Hence, it seems that WSC with unique solutions behave like other tasks in which there is only one possible answer when the target stimulus is processed. In addition, our regression analysis indicated that baseline completion was the only variable that predicted priming. Therefore, in studies on priming, the probability of stem completion should be controlled, as it could cancel out other experimental effects.

The second objective of our research was to compare the results obtained in the WSC task with previous data obtained in a WFC task (Soler et al., 2009). In similar comparative studies, some variables, such as prime-target stimuli presentation format (auditory or visual) and type of stimuli (pictures or words), have been manipulated to determine their effect on priming differences between WFC and WSC. This approach has not produced conclusive results (Roediger et al., 1992; Maki, 1995; Ryan et al., 2001; Mitchell and Bruss, 2003; Jones, 2004). Some authors have proposed that the variety of processes used in experiments, and the differences between the characteristics of the stimuli, may explain the aforementioned discrepant results (Roediger et al., 1992; Gibson, 2012). Therefore, to make a valid comparison of priming in WSC and WFC, we established two requirements for our study. First, the characteristics of the words used in both tasks were fully controlled. The stems used in the WSC task were constructed from the same words used to build the fragments used in the WFC task in the study of Soler et al. (2009). In this way, comparison between the tasks was made with an adequate control of linguistic variables (frequency, number of meanings, familiarity, and length). Second, only unique-solution stems and their corresponding unique-solution fragments were used to make comparisons between tasks in order to avoid the effect of response competition, as the search and selection process of the correct response could be one of the factors underlying the contradictory results obtained with WFC and WSC (Ryan et al., 2001). Additionally, the baseline completion effect on priming was controlled, as stems and fragments had the same moderate baseline average (mean = 0.57).

After controlling the abovementioned potentially confounding variables, we drew the following conclusions in relation to the comparison of WSC vs. WFC: first, correct completion probability of a stem or a fragment seems to depend on the same linguistic characteristics of the words from which the targets are built. Regression analysis showed that word length and familiarity explain baseline completion in the WFC task, as was observed in the regression analysis performed in the WSC task. In both tasks, baseline completion is greater in fragments and stems corresponding to high familiarity and shorter words. Therefore, these two variables should be controlled when WFC and WSC are compared, because they have an effect on baseline. Secondly, in relation with priming, the results of the WFC task indicated that this variable correlates with familiarity, frequency of use and baseline completion of the fragment. These results are similar to those obtained in the WSC task. Priming is also greater when the word used to built fragments and stems has a low frequency, familiarity and baseline completion. Finally, regression results in both tasks showed that baseline completion is the most relevant factor explaining priming. Thus, these results suggest that baseline should also be controlled when WFC and WSC are compared. However, it is relevant to highlight that the linguistic variables that we have considered only explain a relative small amount of variance in priming (20–30%) then, other variables or processes, that could be different across tasks, may play a more important role in priming.

In summary, the present research provides information that may be of interest when selecting stems for implicit memory experiments using WSC. Based on our results, we can affirm that care should be taken in the choice of stems, as they are complex stimuli defined by a set of characteristics, some of which have a clear influence on priming. Such variables include the number of possible solutions and stem baseline completion. In the future, it would be interesting to systematically analyze the effect of these variables on priming using a larger number of stimuli. This would require building a database of Spanish stems, a resource that is currently unavailable, which constitutes a clear limitation. Such a database would provide useful information; for example, more unique-solution stems would be identified, which would allow random sampling of these stimuli when necessary, or information related with the stems baseline completion. Such research is necessary to provide adequate information regarding stimuli when comparing WSC and WFC tasks.

Finally, we must recognize some limitations of this study. First, the number of observations for each stimulus was different; future research should equate them as much as possible. Second, encoding instructions provided to participants in these tasks can influence the cognitive processes explaining priming. It should be noted that our results are based on a particular implementation of the task; prime-target presentation modality was visual and encoding instructions did not emphasize perceptual-orthographic aspects of the stimuli. Future studies should explore if the manipulation of such conditions can have an effect on priming.

### Conflict of Interest Statement

The authors declare that the research was conducted in the absence of any commercial or financial relationships that could be construed as a potential conflict of interest.
